# Molecular characterization of enterotoxigenic *Escherichia coli* toxins and colonization factors in children under five years with acute diarrhea attending Kisii Teaching and Referral Hospital, Kenya

**DOI:** 10.1186/s40794-021-00157-z

**Published:** 2021-12-15

**Authors:** Erick Kipkirui, Margaret Koech, Abigael Ombogo, Ronald Kirera, Janet Ndonye, Nancy Kipkemoi, Mary Kirui, Cliff Philip, Amanda Roth, Alexander Flynn, Elizabeth Odundo, Janeth Kombich, Ibrahim Daud

**Affiliations:** 1 United States Army Medical Research Directorate-Africa/Kenya, Nairobi, Kenya; 2 Walter Reed Army Institute of Research, Silver Spring, USA; 3 University of Kabianga, Kericho, Kenya

**Keywords:** Enterotoxigenic *Escherichia coli*, Toxins, Colonization factors, Acute diarrhea and children

## Abstract

**Background:**

Enterotoxigenic *Escherichia coli* (ETEC) is one of the leading causes of infectious diarrhea in children. There are no licensed vaccines against ETEC. This study aimed at characterizing *Escherichia coli* for ETEC enterotoxins and colonization factors from children < 5 years with acute diarrhea and had not taken antibiotics prior to seeking medical attention at the hospital.

**Methods:**

A total of 225 randomly selected archived *E. coli* strains originally isolated from 225 children with acute diarrhea were cultured. DNA was extracted and screened by multiplex polymerase chain reaction (PCR) for three ETEC toxins. All positives were then screened for 11 colonization factors by PCR.

**Results:**

Out of 225 *E. coli* strains tested, 23 (10.2%) were ETEC. Heat-stable toxin (ST) gene was detected in 16 (69.6%). ETEC isolates with heat-stable toxin of human origin (STh) and heat-stable toxin of porcine origin (STp) distributed as 11 (68.8%) and 5 (31.2%) respectively. Heat-labile toxin gene (LT) was detected in 5 (21.7%) of the ETEC isolates. Both ST and LT toxin genes were detected in 2 (8.7%) of the ETEC isolates. CF genes were detected in 14 (60.9%) ETEC strains with a majority having CS6 6 (42.9%) gene followed by a combination of CFA/I + CS21 gene detected in 3 (21.4%). CS14, CS3, CS7 and a combination of CS5 + CS6, CS2 + CS3 genes were detected equally in 1 (7.1%) ETEC isolate each. CFA/I, CS4, CS5, CS2, CS17/19, CS1/PCFO71 and CS21 genes tested were not detected. We did not detect CF genes in 9 (39.1%) ETEC isolates. More CFs were associated with ETEC strains with ST genes.

**Conclusion:**

ETEC strains with ST genes were the most common and had the most associated CFs. A majority of ETEC strains had CS6 gene. In 9 (39.1%) of the evaluated ETEC isolates, we did not detect an identifiable CF.

## Background

Diarrhea is the second leading cause of death in infants and children in communities with poor sanitation and hygiene. Worldwide there are over 1.3 million deaths every year due to diarrhea. Up to 25% of deaths in children under the age of 5 years living in Africa and south-east Asia are due to diarrhea [1]. A recent study found bacteria to be the most common cause [2] with enterotoxigenic *Escherichia coli* (ETEC) ranked as the 8th leading cause of diarrhea mortality in 2016 among all age groups [3].. Indeed, ETEC is often the first bacterial pathogen that causes diarrhea in infants and children and is responsible for two to five diarrheal episodes during the first 3 years of life [4]. In addition, ETEC causes 80% of diarrhea in travelers from high income countries to low- and middle-income countries [5]. Thus, ETEC remains a major health threat to military soldiers deployed in endemic areas [6].

Enterotoxigenic *E. coli* is identified by its ability to produce plasmid encoded heat-labile (LT) and/or heat-stable (ST) enterotoxins [7, 8]. Based on their host species, ST enterotoxins are further divided into heat-stable enterotoxins of porcine origin (STp) and heat-stable enterotoxins of human origin (STh). STp were originally isolated from domesticated pigs and has been implicated in human disease. STh is only produced by human ETEC isolates [9]. Both of these enterotoxins play a role in disrupting intestinal fluid homeostasis by increasing secretion of fluids and electrolytes through downstream activation of adenylate cyclase (by LT) or guanylate cyclase (by ST) in the small intestinal epithelial cells. When this process exceeds the absorption capacity of the bowel a watery diarrhea is passed [10]. A recent study has shown that LT contributes to ETEC pathogenesis by facilitating the initial adherence and subsequent intestinal colonization of murine and porcine intestinal mucosa. In this study it was reported that ETEC expressed F4ac fimbriae which enabled adhesion to the small intestine epithelial cells [11].

Colonization of the small intestine epithelial cells by ETEC is mediated by means of antigenically diverse thread like appendages (fimbriae) called colonization factors (CF) [7]. These factors are also referred to as colonization factor antigen 1 (CFA/1) or coli surface (CS) antigen [8]. To date, more than 25 CFs have been identified and are classified into three families: (i) CFA/I group which include: CFA/I, CS1, CS2, CS4, CS14 and CS17 (ii) CS5 like group which include: CS5, CS7, CS18 and CS20 and (iii) a distinct group which include: CS3, CS6, CS10, CS11 and CS12 [12]. ETEC can express one or multiple CFs, however, some ETEC strains do not express any known CF [12, 13].

Multiple studies on diarrhea in Kenya have identified and reported ETEC as a major cause of diarrhea in children [14–19]. Due to a lack of capacity at most public laboratories to perform molecular testing, many of these studies do not characterize for STp and CFs in ETEC. Consequently, there are limited data on ETEC CF prevalence in many population demographics, including children under the age of 5 years. To address this knowledge gap, this study was conducted to characterize *Escherichia coli* isolates for ETEC enterotoxins and colonization factors from children under the age of 5 years presenting with acute diarrhea to the Kisii Teaching and Referral Hospital outpatient department.

## Materials and methods

### Study design and site

This was a cross-sectional study nested in an ongoing enteric pathogen surveillance (EPS) study with in Kenya. In the EPS study, parents/guardians provided consent for their children’s participation in the study as well as consent for the storage and future analysis of bacterial isolates. The study was conducted at the United States Army Medical Research Directorate-Africa (USAMRD-A) Microbiology Hub Kericho (MHK) located in Kericho, Kenya.

### Study population

The *E. coli* isolates characterized in this study were isolated from children enrolled in the EPS study protocol. The EPS study enrolls patients of all ages with acute diarrhea and asymptomatic age-matched controls in various outpatient and inpatient hospitals at Kenya Ministry of Health facilities. Stool specimens are transported to the MHK laboratory in Cary-Blair transport media and tested for common bacterial pathogens including *E. coli*, *Vibrio* spp., *Salmonella* spp., *Shigella* spp. and *Campylobacter* spp. by culture and biochemical analysis. Identified bacterial isolates are archived in a repository in 50% glycerol at − 80 °C. *The E. coli* isolates evaluated were isolated from children under the age of 5 years with acute diarrhea enrolled into the EPS study from May 2011 to June 2019 at Kisii Teaching and Referral Hospital (KTRH).

### Inclusion criteria

From the parent EPS, study subject’s inclusion criteria included: patients with acute diarrhea, defined as having three or more loose/watery stools within a 24-h period, lasting less than 14 days and have not taken antibiotics to treat the diarrhea prior to visiting the hospital. For this study the inclusion criteria were: *E. coli* isolates from case subjects under the age of 5 years and viable upon subculture.

### Exclusion criteria

The EPS study excluded individuals with chronic diarrhea (diarrhea lasting more than 2 weeks), those who had taken antibiotics and those unwilling to provide informed consent or give a stool specimen. For this study, additional exclusion criteria were: *E. coli* isolates from case subjects above 5 years of age and those not viable upon subculture.

### Sample size and sampling technique

The sample size for this study was calculated using a single population proportion formula [20] with 95% confidence interval and 5% margin of error. The proportion of ETEC was taken to be 16.2% according to a study done in Kenya [14]. Thus, the minimum sample size was 208 children with *E. coli* isolated from them. We added 17 more children with *E. coli* to allow the study to cover an 8-year period. As a result, a total of 225 children each with *E. coli* were included in this study. Random sampling was used to select the archived *E. coli* isolates from the freezer using a register that is maintained for the EPS protocol.

### Laboratory analysis

#### *Escherichia coli* isolates revival

The *E. coli* isolates utilized in this study were already identified using biochemical tests including Gram staining, catalase, oxidase, indole test, triple sugar iron agar and Voges Proskauer tests. The archived *E. coli* isolates were randomly selected from the MHK repository. The vial of the frozen bacterial stock was removed and inoculum picked using an inoculating needle, streaked on MacConkey agar (Becton Dickinson, Franklin Lakes, NJ, USA) and incubated overnight at 37 °C. All the *E. coli* isolates were viable upon subculture. *E. coli* lactose-fermenting colonies on MacConkey agar were processed for DNA extraction.

#### Bacterial DNA extraction

Bacterial DNA template for ETEC enterotoxins and CF screening was extracted by boiling method [21] with slight modifications. Briefly, 5–10 *E.coli* colonies from MacConkey agar plates were picked with a sterile loop and suspended in 200 μl of sterile nuclease free water and vortexed for 30 s. The bacterial suspensions were boiled on a heat block at 100 °C for 20 min and centrifuged at 12,000 rpm for 10 min. The supernatant containing DNA was picked and stored at − 20 °C prior to detection of ETEC toxins and CFs.

#### Detection of ETEC enterotoxins genes

ETEC virulence (*eltB1*, *estA1* and *estA2*–4) genes were screened by an end point PCR using validated DNA primers (Table 1). The 25 μl PCR reaction mixture contained 12.5 μl of 2X DreamTaq Green PCR Master Mix (Thermo Scientific, Waltham, MA, USA), 0.5 μl (30 μM) each of forward and reverse primers for *eltB1*, *estA1* and *estA2–4* genes, 4.5 μl of nuclease free water and 5 μl of DNA template. The PCR reaction was performed on a Veriti™ thermocycler (Applied Biosystems, Foster City, CA, USA) with an initial denaturation of 95 °C for 2 min, followed by 30 cycles of amplification (95 °C for 1 min, 58 °C for 30 s and 72 °C for 1 min), 1 cycle at 95 °C for 1 min, 58 °C for 30 s and a final extension step at 72 °C for 10 min. Positive and negative controls were ETEC strain H10407 and nuclease-free water respectively.

**Table 1:** Primers used to detect ETEC enterotoxins and colonization factors [21]

ETEC toxin/colonization factor	Target gene	Primer name	Sequence (5′ to 3′)	Amplicon size (bp)
LT	*eltB1*	LThF1	CATAATGAGTACTTCGATAGAGGAAC	402
		LThR1	GAAACCTGCTAATCTGTAACCATCC	
STp	*estA*1	STpF1	ATGAAAAAGCTAATGTTGGCA	239
		STpR1	TTAATAACATCCAGCACAGGCA	
STh	*estA*2–4	estA2–4F	AATTGCTACTATTCATGCTTTCAGGAC	133
		estA2–4R	TCTTTTTCACCTTTCGCTCAGG	
CFA/I	*cfaB*	mpcr gp1F	TGAGTGCTTCWGCAGTAGAGA	204
		CFA1R	CAGCAAGTTTAACAATTACTTTTTTAGT	
CS4	*csaB*	mpcr gp1F	TGAGTGCTTCWGCAGTAGAGA	300
		CS4R	AAGTCACATCTGCGGTTGATAGAG	
CS14	*csuA1*	mpcr gp1F	TGAGTGCTTCWGCAGTAGAGA	357
		CS14R	TACTATTCGAAACACCTGCCG	
CS6	*cssB*	CssBCS6F	GGAGTGGTAAATGCAGGAAACT	416
		CssbCS6R	GTA CCA GAC GAA TAT CCG CTA TTA	
CS3	*cstA*	CS3F1	GGTCTTTCACTGTCAGCTATGAGTT	136
		CS3R1	TAATGTTAAATTATCCTGAGGAGCC	
CS5	*csfA*	CS5F1	GCGTGACACGTCAGCTAATATAAAC	235
		CS5R	GGCATTCATATCAATAGAAATATGAGAC	
CS7	*csvA*	CS7F	TGCTCCCGTTACTAAAAATACG	418
		CS7R	GGCATTCATATCAATAGAAATATGAGAC	
CS2	*cotA*	CS2F	TCTGCTCGTATCAATACCCAAGTT	140
		CS2R	GTGCCAGCGAATGAAACCTCTAAA	
CS17/19	*csbA* and *csdA*	mpcr GP3	ACTCTRTCGCATTAACCTATTCT	169
		CS17_19R	GTCACTTTCATCGGAATTTGCGAG	
CS21	*IngA*	CS21F	TATGAGCCTKCTGGAAGTYATCAT	292
		CS21R	GTTATTACGCACTTCGTCTGGT	
CS1/PCF071	*csoA*	mpcr gp3	ACTCTRTCGCATTAACCTATTCT	334
		CS1_PCFO71R	CCCTGATATTGACCAGCTGTTAGT	

The ETEC CFs screening was performed in three separate PCR master mixes: master mix I for detection of CFA/I, CS4, CS6 and CS14 genes, master mix II for detection of CS3, CS5 and CS7 genes and master mix III for detection of CS2, CS17/19, CS21 and CS1/PCF071 genes

#### Detection of colonization factors genes

*E. coli* isolates positive for one or multiple enterotoxins were further screened for detection of 11 colonization factors using gene-specific DNA primers (Table 1). Each PCR reaction final volume was 25 μl containing 12.5 μl of Dream Taq Green PCR Master Mix (Thermo Scientific, Waltham, MA, USA), 0.5 μl of forward and reverse primer (30 μM), 3.5 μl of nuclease free water and 5 μl of DNA template for master mix I and III. For master mix II, 4.5 μl was used instead of 3.5 μl of nuclease free water. The PCR reactions were performed on a Veriti™ thermocycler (Applied Biosystems, Foster City, CA, USA) with an initial denaturation of 95 °C for 2 min, followed by 30 cycles of amplification (95 °C for 1 min, 60 °C for 30 s, 72 °C for 1 min), one cycle of 95 °C for 1 min, 60 °C for 30 s, and 72 °C for 10 min. Positive control strains were provided by the Naval Health Research Center (San Diego, CA, USA) and included: H10407 (*cfaB*), BANG10-SP (*csaB* and *cssB*), WS3294A (*csuA1*) B2C (*cstA*, *cotA* and *IngA*), ETEC8/11(*csfA*), D022 (*csvA*), E24377A (*csoA*) and WS0115A (*csbA-csdA*). Nuclease-free water was used as negative control.

#### Gel electrophoresis analysis

The PCR amplicons were separated by electrophoresis on 2% agarose gel (Sigma Aldrich, St. Louis, MO, USA) stained using gel red (Biotium, San Francisco, CA, USA) and visualized under UV light using iBright gel documentation system (Applied Biosystems, Foster City, CA, USA).

#### Data analysis

The frequency of the type of enterotoxins and CFs detected was calculated. GraphPad Prism version 8.3 was used for statistical analysis with Fisher’s exact test used for comparison between groups where appropriate. Differences were considered statistically significant if the *P*-value was less than 0.05.

## Results

### Distribution of children across the age groups

Of the 225 children whose *E. coli* isolates were analyzed in this study, 39 (17.3%), 44 (19.6%), 50 (22.2%), 61 (27.1%) and 31 (13.8%) were aged 1–12 months, > 12–24 months, > 24–36 months, > 36–48 months and < 48- < 60 months respectively (Table 2). The mean age of the study participants providing the randomly selected archived *E. coli* isolates was 31 months.

**Table 2:** Age distribution of children with acute diarrhea associated with ETEC

Age (months)	Number (%) children with ***diarrhea***	Number (%) of ETEC positive children	***p***-value
1–12	39 (17.3)	4 (10.3)	0.978
> 12–24	44 (19.6)	5 (11.4)	
> 24–36	50 (22.2)	4 (8.0)	
> 36–48	61 (27.1)	7 (11.5)	
> 48- < 60	31 (13.8)	3 (9.7)	
Total	225	23 (10.2)	

A majority of the *E. coli* isolates were isolated from children aged between > 36–48 months old

### Distribution of ETEC across the age groups

Of the 225 *E. coli* isolates characterized, 23 (10.2%) were positive for ETEC. The fraction of ETEC isolates were similar in each age group with a majority detected in children aged > 36–48 months old. (Table 2). There was no significant difference in the distribution of ETEC across age groups (*p* = 0.978).

### Detection of ETEC toxins and CFs across the age groups

Heat stable toxin (ST) gene was the most common detected in 16 (69.6%). Heat labile toxin (LT) gene was detected in 5 (21.7%) of the ETEC isolates. Both ST and LT toxins genes were detected in 2 (8.7%) of the isolates. Majority 5 (31.25%) of ETEC with ST gene were detected in children aged > 36–48 months old whileETEC LT gene were equally detected 1 (20%) across all the age groups. ETEC with both LT/ST genes were equally detected 1 (50%) in children aged < 12–24 months and > 36–48 months.

We detected CF genes in 14 (60.9%) of the ETEC isolates. CS6 6 (42.9%) was the most common followed by a combination of CFA/+CS21 genes at 3 (21.4%). CS14, CS3, CS7 and a combination of CS5 + CS6, CS2 + CS3 genes were detected equally in 1 (7.1%) ETEC isolate each. CFA/I, CS4, CS5, CS2, CS17/19, CS1/PCFO71 and CS21 genes were not detected. Each age group had at least one CF detected from the ETEC isolates. Out of the 11 CFs we screened for we did not detect CF genes in 9 (39.1%) ETEC isolates.

### Association of CFs with toxin types

The association of CFs genes differed by ETEC toxin. More CF genes were detected in 11 (78.6%) ETEC- ST while 2 (14.3%) and 1 (7.1%) CF were detected in ETEC-LT and ETEC-LT/ST respectively. In a majority of ETEC- ST we detected CS6. In ETEC -LT, we detected only CS6 and CS7 while in ETEC-LT/ST we detected only CS3. Among the 9 (39.1%) ETEC isolates that lacked an identifiable CF, 3 (60%) were ETEC-LT, 5 (44.5%) were ETEC-ST and 1 (50%) were ETEC -LT/ST.

## Discussion

Enterotoxigenic *E. coli* is a major cause of diarrhea in children below 5 years of age and travelers in less developed and developing countries. In our study, the proportion of ETEC in children under the age of 5 years with acute diarrhea positive for *E.coli* was 10.2%. This finding is close to the proportions reported in a study in Machakos, Kenya [13] and in Bolivia [22] at 11 and 9.2% respectively. Two studies in Indonesia reported higher proportions of 19% [7] and 14.9% [23] while Bangladesh reported 14% [24]. The variation between our finding and these studies could be due to the differences in primers used, geographical factors, target population and number of samples tested.

In our study, we identified that the ST as the most common toxin gene, detectedin 16 (69.6%) of ETEC strains followed by LT detected in 5 (21.7%) while 2 (8.7%) had both LT and ST toxins genes. These findings are consistent with a study in Indonesia where ST toxin was the most common followed by LT while LT/ST genes were the least [23]. A similar finding was also reported in a study across four regions of the world and the Global Enteric Multicenter Study where ST was the most frequent toxin [25, 26]. However, these findings are not in agreement with reports of a study in Nigeria where ST and LT toxins were detected equally among the ETEC isolates [8]. Additionally, a study in Bolivia reported that LT was the most common toxin followed by LT/ST and the least was ST. [22].

We detected CFs genes in 60.9% of the ETEC strains similar to a study in Peru where it was detected at 65% [27] but lower than 80% reported by Kharat et al. [25]. Our finding is higher than the finding in studies carried out in Nigeria (43.7%), Brazil (50%) and Indonesia (18.2%) [7, 8, 28]. CS6 was the most detected CFs in our study. This is consistent with finding from a study by [Simuyandi et al. [12], Qadri et al. [24], Taxt et al. [29], Blackburn et al. [30]] where CS6 was the most common CF but contrasts to a study in Kenya where the CFA/I was the most common followed by CS6 [13]. In Bolivia CFA/I was the most common followed by CS17 [22]. A study by Kharat et al. [25] reported that CS21 was the most common followed by CS6. The difference between our findings and those from other studies may be due to the difference in the expression of CFs by ETEC in different geographical regions, the difference in the laboratory methods/ primers employed in the identification of CFs [28]. Additionally, loss of plasmids carrying the CFs could be another reason for the differences [13].

In our study 9 (39.1%) of ETEC lacked an identifiable CF. This observation is consistent with the finding in Kenya [13] that reported 36.7% of ETEC without an identifiable CF. Studies in Egypt reported approximately 50% of ETEC isolates lacked an identifiable CF [31, 32]. The lack of an identifiable CF could be explained by the following reasons; loss of plasmid harboring the CFs genes due to repeated sub culturing or long-term storage; down regulation of CF genes; a mutation within the genetic locus; expression of a CF not covered by the primers used in the PCR panel and CFs that are unknown and have not been classified [7, 13, 23, 28]. The lack of CF has been reported to be mainly related to LT strains [8, 13, 27, 32] which is consistent with our results where 60% of the LT strains lacked a detectable CF. However, some studies have reported that CFs is almost equally associated with LT and ST-positive ETEC strains [8, 28].

## Conclusion

This study documents the detection of ETEC in children under the age of 5 years with acute diarrhea from a government-run community hospital in Kenya. ETEC producing ST was the most common strain in children with acute diarrhea. CS6 was the most common CF and that more CFs were detected from the ETEC strains producing ST. These results, and their variation from others published in other low- and low- to middle-income countries, emphasize the value of continuous identification of ETEC toxins and colonization factors in different regions and populations in Kenya so as to better inform the prevalence and vaccine development strategies. Development of an ETEC vaccine is dependent on an in-depth understanding of toxins and CF distribution. Our results provide useful information on ETEC virulence factors both toxins and colonization factors that can be considered as among the targets in the development of an ETEC vaccine.

### Limitations of the study

While this is the first report to characterize ETEC for STp and CFs genes from KTRH diarrheal samples from children under five, in this study we did not test for non-classical virulence genes of ETEC and all the CFs that have been described. We did not also look into antibiotic resistance patterns of the ETEC detected. However, the results do follow trends seen in other studies.

### Future research

There is need to carry out further research to test for non-classical virulence genes of ETEC to determine how ETEC strains that lack an identifiable CFs are able to cause diarrhea especially in children under 5 years. Also, we recommend that future studies should include asymptomatic children to enable a more conclusive finding on the burden of ETEC in terms of toxins and CFs on children below the age of 5 years.

## Data Availability

All the data generated or analyzed during this study are included in this published article.

## Abbreviations

CF: Colonization factor
CFA: Colonization factor antigen
CS: Coli surface
°C: Degree centigrade
DNA: Deoxyribonucleic acid
*E. coli*: *Escherichia coli*
EPS: Enteric Pathogens Surveillance
ETEC: Enterotoxigenic *Escherichia coli*
KEMRI-SERU: Kenya Medical Research Institute Scientific Ethical Review Unit
KTRH: Kisii Teaching and Referral Hospital
LT: Heat labile toxin
MHK: Microbiology Hub Kericho
PCR: Polymerase chain reaction
rpm: Revolution per minute
*spp*: Species
ST: Heat stable toxin
STh: Heat stable toxin of human origin
STp: Heat stable toxin of porcine origin
USAMRD-A/K: United States Army Medical Research Directorate Africa/Kenya
μl: Microliter
UV: Ultraviolet
%: Percentage
